# Viscosupplementation in the treatment of osteoarthritis

**DOI:** 10.1186/ar3717

**Published:** 2012-03-08

**Authors:** Alberto Migliore, Francesca Giovannangeli

**Affiliations:** 1 UOS of Rheumatology, S. Peter Hospital, Rome, Italy

## 

Osteoarthritis is the most common joint disease and a major cause of disability. The changes in the lubricating properties of synovial fluid lead to significant pain and function loss. The injection of hyaluronic acid (HA) into the joints, called Viscosupplementation, improves the biochemical properties of synovial fluid (SF) in osteoarthritis (OA) joints. The clinical effect is pain relief and someone has hypothesized a disease modifying activity . Intra-articular (IA) hyaluronan therapy has been used in the treatment of symptoms associated with OA of the knee and other joints with a very favourable safety profile. The high concentration of HA in SF is essential for normal joint function. Balazs and Denlinger were the first to suggest that the favourable effects of IA HA therapy in OA was related to the SF rheological properties restoration. Many subsequent studies have supported that IA HA therapy could be a symptom-modifying approach. The hypothesis that HA could bind specific receptors is now widely accepted. The cluster determinant 44 (CD44), the intracellular adhesion molecule-1 (ICAM-1) and hyaluronate-mediated motility receptor (RHAMM) expressed in many cells, are some of those implicated in the process. The binding of HA with its specific receptors has been reported to trigger various intracellular signals such as cytokine release and stimulation of cell cycle proteins. The consequences of these interactions is to stimulate cell functional activities such as cell migration and proliferation. Presently, there are several different HA products with different molecular weight available for injection. Several meta-analysis have reviewed clinical trials published in the last years using different HA preparation that support the benefit and safety of repeated treatment with IA HA. Many trials indicate that sodium hyaluronate is well tolerated and as effective after multiple courses of treatment as it is after a single course. Other informations state that viscosupplementation is an effective treatment for patients with knee OA who have ongoing pain or are unable to tolerate conservative treatment or joint replacement. Viscosupplementation appears to have a slower onset of action than intra-articular steroids, but the effect seems to last longer. Viscosupplementation is an effective treatment for OA of the knee with beneficial effects on pain, function and patient global assessment at different post injection periods but especially at the 5 to 13 week with a dramatic effect on the reduction of NSAIDs monthly consumption. In some analyses viscosupplements were comparable in efficacy to systemic forms of treatment, with more local reactions but fewer systemic adverse events. In other analyses HA products had more prolonged effects than IA corticosteroids. Overall, the aforementioned analyses support the use of the HA class of products in the treatment of knee OA. In one systematic review intra-articular hyaluronic acid has not been proven clinically effective and may be associated with minimal and transient risk of adverse events. The heterogeneity of these studies limits definitive conclusions on different treatment regimen with different molecular weight HA but, in our opinion, we think that generally HA products may determine a reduction of symptoms by 40%. Few data exists in literature about the viscosupplementation of hip OA, the second most common site of the disease after the knee. The intra-articular injection of the hip is not as easy as for the knee, mainly for anatomical features of the joint and the proximity of "sensitive" structures such as the femoral artery and nerves. Even though hip injection may be performed "blindly", nevertheless failure rate is significant and when using a slow absorbing viscosupplement like hyaluronan the potential local complications may jeopardize the therapeutic benefit. For such reasons, it has been suggested to perform intra-articular injection of the hip under radiological or ultrasound control. Although several ultrasound guidance techniques were available we developed a personal one using an antero-superior approach. The efficacy data, presents in literature, underline significant improvement in pain and function as has been shown in the knee OA despite the small size of the sample and the short period of observation have not allowed to point out a change in the natural history of the disease. In order to confirm these promising data large scale double blind controlled trials must be carried out. Despite recent progress, many unresolved issues require further study. These include the necessity to determine the relative economic efficacy of viscosupplementation, explore the therapeutic effectiveness and safety, clearly define the relation between molecular weight and clinical effectiveness of the different hyaluronan products, establish optimal dosing regiments and assess potential synergistic or additive effects with other modalities such NSAIDs and SYSADOA. If hyaluronan derivatives are eventually proven to have clear disease-modifying effects, it may then be reasonable to consider their use early in cartilage injury or disease, before the onset of syntomatic OA. Additional well-designed randomized controlled trials with high methodological quality are needed to resolve the continued uncertainty about the therapeutic effects of different types of hyaluronic acid products on osteoarthritis.

